# A Mindfulness-Based Intervention to Alleviate Stress From Discrimination Among Young Sexual and Gender Minorities of Color: Protocol for a Pilot Optimization Trial

**DOI:** 10.2196/35593

**Published:** 2022-01-14

**Authors:** Stephanie H Cook, Erica P Wood, Nicholas Mirin, Michelle Bandel, Maxline Delorme, Laila Gad, Olive Jayakar, Zainab Mustafa, Raquel Tatar, Shabnam Javdani, Erin Godfrey

**Affiliations:** 1 Department of Social and Behavioral Sciences School of Global Public Health New York University New York, NY United States; 2 Department of Biostatistics School of Global Public Health New York University New York, NY United States; 3 New York University New York, NY United States; 4 Healthy Minds Innovations Madison, WI United States; 5 Department of Applied Psychology Steinhardt School of Culture, Education, and Human Development New York University New York, NY United States

**Keywords:** sexual and gender minorities, racial/ethnic minorities, mindfulness, mobile phone

## Abstract

**Background:**

Young sexual and gender minorities (SGMs) of color may face unique experiences of discrimination based on their intersectional positions (eg, discrimination based on both racial or ethnic identity and sexual identity). Emerging evidence suggests that mindfulness practices may reduce stress from discrimination and improve overall well-being among young SGM. Moreover, the omnipresence of smartphone access among racial or ethnic and sexual minority communities provides a method through which to administer mindfulness-based interventions among young SGMs of color.

**Objective:**

This paper outlines the protocol of the Optimizing a Daily Mindfulness Intervention to Reduce Stress from Discrimination among Young Sexual and Gender Minorities of Color (REDUCE) study, a pilot optimization trial of a smartphone-based mindfulness intervention that was developed in conjunction with the Healthy Minds Program (HMP) with the aim of reducing stress from discrimination among young SGMs.

**Methods:**

In total, 80 young (ages 18-29 years) SGMs of color will be enrolled in the study. The HMP is a self-guided meditation practice, and participants will be randomized to either a control condition or an intervention that uses a neuroscience-based approach to mindfulness. We will use the multiphase optimization strategy to assess which combination of mindfulness interventions is the most effective at reducing stress from discrimination among young SGMs of color. A combination of mindfulness-based meditation intervention components will be examined, comprising mindfulness-based practices of awareness, connection, and purpose. Awareness refers to the practice of self-awareness, which reduces the mind’s ability to be distracted and instead be present in the moment. Connection refers to the practice of connection with oneself and others and emphasizes on empathy and compassion with oneself and others. Purpose encourages goal-making in accordance with one’s values and management of behavior in accordance with these goals. In addition, we will assess the feasibility and acceptability of the HMP application among young SGMs of color.

**Results:**

The REDUCE study was approved by the Institutional Review Board of New York University, and recruitment and enrollment began in the winter of 2021. We expect to complete enrollment by the summer of 2022. The results will be disseminated via social media, journal articles, abstracts, or presentations, as well as to participants, who will be given the opportunity to provide feedback to the researchers.

**Conclusions:**

This optimization trial is designed to test the efficacy, feasibility, and acceptability of implementing an application-based, mindfulness-based intervention to reduce stress from discrimination and improve well-being among young SGMs of color. Evidence from this study will assist in the creation of a sustainable, culturally relevant mobile app–based mindfulness intervention to reduce stress from discrimination among young SGMs of color.

**Trial Registration:**

Clinicaltrials.gov NCT05131360; https://clinicaltrials.gov/ct2/show/NCT05131360

**International Registered Report Identifier (IRRID):**

DERR1-10.2196/35593

## Introduction

### Minority Stress and the Health of Sexual and Gender Minorities

The term sexual and gender minority (SGM) comprises a broad range of identities related to sexual orientation and gender identity including, but not limited to, lesbian, gay, bisexual, and transgender individuals [1]. Many studies have documented that SGMs are faced with a multitude of health disparities, particularly within the emerging adulthood period (ie, ages 18-29 years) [2,3]. In particular, depression, anxiety, and suicidality continue to be disproportionately high throughout the emerging adulthood period among SGMs relative to older SGMs and non-SGMs [3]. Moreover, emerging adulthood is a key developmental period in which interventions designed to buffer the negative effects of stress on health may be particularly relevant, as emerging adulthood is characterized by rapid changes in psychological, social, and familial environments (eg, college) that greatly affect the development and maintenance of stress-coping mechanisms [4].

One pathway through which mental health disparities are posited to arise among SGMs is through sexual minority stress [5]. The sexual minority stress theory posits that sexual minority individuals experience many distal and proximal stressors related to the negative social valuation of sexual minority identity, resulting in exacerbated stress beyond the levels that people generally experience [5]. Over time, individuals may internalize minority stressors (eg, internalized homophobia), which in turn may contribute to poor mental health among SGMs [5]. Furthermore, SGMs who experience intersectional discrimination (eg, gender-based, racial- or ethnic-related, and sexual orientation–related discrimination) are more likely to report poorer mental health as compared with their White SGM and non-SGM counterparts [6-8]. Although the sexual minority stress theory also suggests that endemic structural-level processes contribute to the perpetuation of poor mental health among SGMs, it is imperative that interventions not only focus on reducing mental health inequalities at the societal level but also focus on reducing the *impact* of discrimination in the everyday lives of SGMs, particularly SGMs of color.

Researchers using daily diary methodologies have found that emerging adulthood SGMs who report more daily discriminatory experiences have more negative mood, poorer emotional states, and report more depressive symptoms and suicidal ideation on average than those who report fewer daily discriminatory experiences [9]. In addition, young SGMs of color who experience intersectional discrimination based on race or ethnicity, gender identity, or sexual orientation report more negative mood than young White SGMs [9].

### Mindfulness and SGM Mental Health

Research suggests that increasing the practice of mindfulness may be useful for reducing the impact of daily stress on poor mental health among emerging adults who experience discrimination [10]. Mindfulness meditation is a practice that focuses on an individual’s attention on the present moment, leading to reduced levels of stress [11]. Web-based mindfulness interventions among SGMs have been associated with lower levels of perceived stress compared with baseline readings in both men and women, as well as reduced sexual minority stress in SGM women [12]. Mindfulness interventions can also lead to increased individual resilience toward discriminatory experiences [13,14]. By allowing the SGM individual to disengage with the discriminatory experience by focusing on the present moment, negative feelings may be less likely to occur, which can lead to improved psychological well-being [13]. Past research provides evidence that mindfulness acts as a protective factor against the negative psychological effects of school-based victimization based on sexual orientation and age-based discrimination [14,15]. In particular, higher levels of mindfulness buffered the association between discrimination events and negative psychological outcomes, such as anxiety and depression [14,15]. Although there is a critical number of observational and intervention studies showing the effectiveness of mindfulness in reducing stress from discrimination and promoting well-being [16], the key features of mindfulness interventions that are the most effective, efficient, and scalable remain unclear. Thus, an approach to empirically examine the key features of mindfulness that are effective in reducing stress from discrimination among SGMs is imperative for creating culturally relevant and impactful well-being interventions for diverse populations of SGMs. In particular, there are three specific mindfulness-based practices, that is, awareness, connection, and purpose, that may lead to reductions in levels of stress and increased well-being.

*Awareness* through meditation promotes well-being through self-awareness, reducing the mind’s ability to be distracted and instead be present in the moment, not allowing the mind to wander to negative thoughts that cause stress. Practicing awareness brings you to the present moment and allows you to become *aware* of your thoughts. Should the thoughts become negative, awareness practice teaches individuals to let those thoughts wander without judgment. Furthermore, this practice teaches people to be present in the *current moment* rather than in thoughts regarding the past or the future [17]. The impact of awareness-based practice on mental well-being is supported by the overarching research literature [11,18]. For example, 1 study found that those who did not engage in awareness-based practices reported lower levels of happiness as compared with those who engaged in awareness-based practices [11]. Moreover, in another study of 717 students, Parto and Besharat [19] found that awareness was positively associated with mental well-being in their study sample. Thus, there is evidence to support that awareness is associated with positive mental health outcomes.

*Connection* through meditation supports empathy, compassion, and kindness in daily activities. The practice of connection emphasizes connections with oneself and with others. The mindfulness-based practice of connection with oneself highlights the need to be aware of oneself and one’s emotions or feelings, to accept oneself owing to this awareness, and to orientate one’s behaviors in conjunction with this awareness of oneself [20]. Moreover, mindfulness-based practices can also foster connections with others through *compassion* meditation, which begins with attention training and mindfulness, and goes on to contemplative practices with the goal of highlighting connection with others and self-compassion. Research shows that mindfulness-based self-connection is associated with well-being. Indeed, 1 study found that mindfulness predicted self-connection, which in turn was associated with increased well-being [20]. Moreover, previous work demonstrates that engagement in *compassion* meditations based on mindfulness and compassion is associated with reduced psychological distress and lower depressive symptoms [21,22].

*Purpose* through meditation encourages daily meaning in life through the encouragement of goal-making and behavior management in accordance with these goals [23]. The mindfulness-based practice of purpose allows emotions to be better regulated when an individual acknowledges the meaning in their life, leading to clearer values and persistence in the face of adversity. This practice guides overarching goals and daily behaviors by offering direction to oneself in alignment with one’s goals and needs [23]. Much research documents the benefits of committing to the mindfulness-based practice of purpose. For instance, a systematic review found that meaning in life (ie, purpose) was associated with better overall physical health [24]. In addition, in a longitudinal study, Disabato et al [25] found in a sample of 797 adults that meaning in life predicted decreases in depressive symptoms over a 6-month period.

### Conceptual Framework

**Figure 1:**
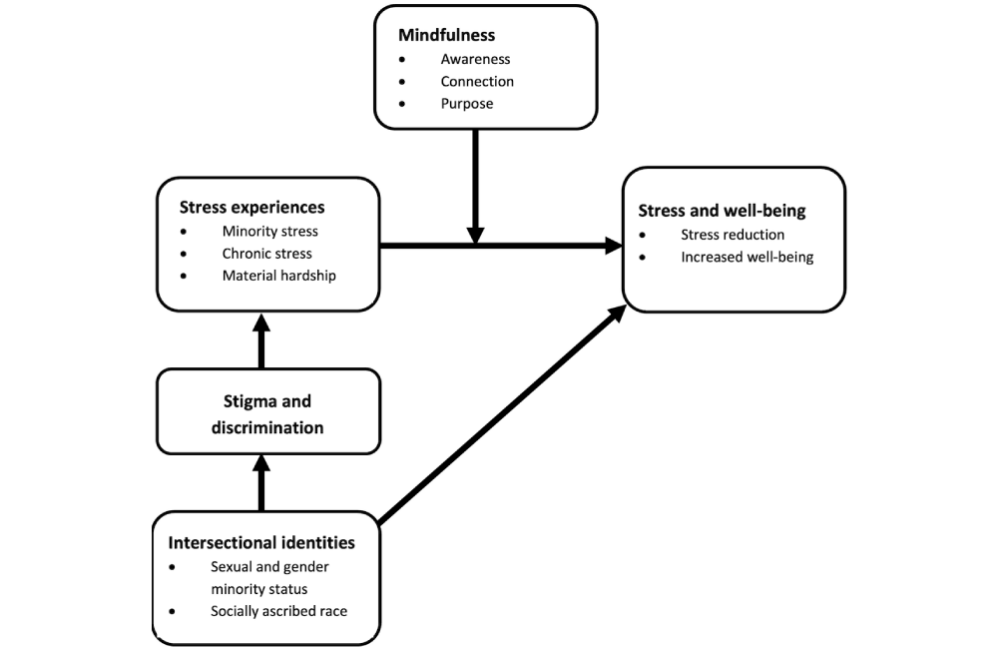
Conceptual framework.

Figure 1 displays the study’s overarching conceptual framework. This framework describes how intersectional identities may lead to increased levels of stress and reduced well-being among young SGMs of color. SGMs of color possess multiple marginalized identities (ie, sexual or gender minority status and race or ethnicity). The possession of multiple marginalized identities, in turn, may lead to exposure to stigma and discrimination at the intersection of these identities (eg, they may experience discrimination based on both their gender identity and race or ethnicity). Exposure to stigma or discrimination may lead to increased stress levels in the form of minority stress, which may interact with other forms of stress (eg, chronic stress and financial hardship), which, in turn, may lead to poor mental health outcomes, including increased perceived stress and decreased well-being. However, the practice of mindfulness in the domains of awareness, connection, and purpose may buffer the negative effects of minority stress on perceived stress and well-being among young SGMs of color such that those who engage in these practices may experience improved mental health in the form of reduced perceived stress and increased well-being.

### The Multiphase Optimization Strategy Framework

The primary goal of this study is to use an innovative optimization design (ie, the multiphase optimization strategy [MOST]) to evaluate different components of mindfulness interventions and the interaction between the components to build an effective, efficient, and scalable intervention to reduce daily stress from discrimination among SGMs of color. MOST is a systematic method for identifying the optimized combination of intervention components before testing an intervention in a resource-intensive randomized controlled trial (RCT) [26,27]. The MOST consists of three stages: preparation, optimization, and evaluation of the optimized intervention in an RCT. In this study, participants will be randomized into 1 of 8 conditions, and interventions will be administered using the MOST design. The participants will be randomized to at least one of the 3 intervention components of awareness, connection, and purpose for this optimization study, which comprises the 8 conditions. Each of these components is known to be effective in reducing stress and promoting well-being among populations that experience high rates of discrimination (eg, SGMs) [12-14]. These components will be administered via the Healthy Minds Program (HMP) app with the goal of examining which combination of components most effectively reduces stress from discrimination among racial or ethnic SGMs.

### Study Objectives

In this study, the goal is to use the MOST to evaluate different components of mindfulness interventions and the interaction between the components to build an effective, efficient, and scalable intervention to reduce daily stress from discrimination among SGMs of color. The overall objective of this study is to identify which combination of the three components (ie, purpose, connection, and awareness) meaningfully contributes to improvement in the primary outcomes of interest among SGMs, which are perceived stress reduction (ie, one’s feeling about how stressed they are at a current moment) and improved well-being (ie, the state of feeling comfortable, happy, and healthy).

The aims of this study are as follows: (1) to assess the efficacy of the intervention components separately and in combination on mean change in stress and well-being among SGMs and (2) to determine the most effective intervention, based on the different combinations of the intervention components, which leads to the greatest reductions in perceived stress and the greatest improvement in mental well-being among SGMs. The 5-day period has been used in previous studies and demonstrates that mindfulness-based practice is effective at reducing stress over this brief period [28]. Furthermore, as a secondary aim, we will examine the feasibility and acceptability of the Reduce Stress from Discrimination among Young Sexual and Gender Minorities of Color (REDUCE) study. Feasibility and acceptability will be measured via retention rates and debriefing interviews, where we ask participants about their experiences with the study.

## Methods

### Setting and Participants

REDUCE is a single-site, pilot optimization study with the primary goal of assessing which component or components of a mindfulness-based smartphone app, the *HMP*, most effectively reduces the effects of stress from discrimination among young SGMs of color. The study will use the MOST [26,27] framework to examine which combination of the well-being intervention components (ie, awareness, connection, and purpose; see the Intervention section) leads to the most optimal behavioral intervention. The *most optimal* behavioral intervention in this context is defined as one that yields the greatest improvement in mental health outcomes (ie, perceived stress and mental well-being) [29].

This pilot study will consist of 80 young SGMs (ie, between the ages of 18 and 29 years) who identify as a racial or ethnic minority (eg, Black or Hispanic). Participants will be randomized to 1 of 8 conditions (including a control condition; explained in detail in the Study Procedures section). The HMP app was developed by Dr Richard Davidson and others at the University of Wisconsin-Madison’s Center for Healthy Minds in affiliation with the nonprofit Healthy Minds Innovations [30]. The HMP smartphone app is compatible with both the iPhone Operating System (iOS) and Android. The REDUCE study will be web- and smartphone-based, with study staff communicating with participants through Zoom (Zoom Video Communications), text message, phone, or email, depending on participant preference.

Participation in the study will last for 6 days, consisting of a web-based baseline survey (day 1), 5 days of self-guided practices completed through the HMP app (days 2-6), and 5 days of web-based daily diaries (days 2-6). In addition, we will collect data pertaining to feasibility and acceptability through a debriefing interview to be completed after completion of the 6-day study protocol. The Institutional Review Board of New York University at Washington Square has reviewed and approved this study (IRB-FY2020-4338).

### Eligibility Criteria

Young SGMs of color who are aged between 18 and 29 years and who live in the New York metropolitan area will be eligible to participate in the study. The eligibility criteria were as follows: (1) be aged between 18 and 29 years; (2) must identify as a sexual minority (gay, bisexual, etc); (3) must identify as an underrepresented racial or ethnic minority (eg, Black or Hispanic); (4) must have an active smartphone and be able to access the smartphone 7 days a week between 6 PM and 6 AM the next morning; (5) must be willing and able to receive up to 6 text messages or emails per day; (6) must have consistent internet access 7 days a week between 6 PM and 6 AM; (7) must have the ability to understand, read, and speak English; (8) must reside in the New York metropolitan area; and (9) must be willing to provide written informed consent. We will exclude individuals who do not meet any of the eligibility criteria (eg, White participants).

### Study Procedures

#### Recruitment

This study will focus on SGMs of color at the intersection of multiple marginalized identities (eg, race or ethnicity and sexual orientation). Participants residing in the New York metropolitan area (*n=*80) will be recruited using offline and web-based techniques, including the posting of flyers around New York City–based campuses (eg, New York University [NYU], Columbia) and web-based listservs aimed at reaching young adult lesbian, gay, and bisexual (LGB) populations.

#### Screening

Interested persons will be directed to a web-based survey screener hosted on REDCap [31,32]. This web-based survey screener will provide a brief overview of the study to prospective participants. Prospective participants will be provided with contact information (ie, phone number and email) for both the principal investigator and laboratory at large, should they have any questions regarding the study objectives or protocol. Only those who are deemed eligible by the study screener (see the *Eligibility Criteria* section) will be contacted by the study staff. Ineligible responses will be kept on a file with a justification indicating why they were not eligible for the study.

### Consent or Enrollment

If a prospective participant screens eligible through the web-based study screener, they will be contacted by trained study staff via email to schedule their informed consent session, which will take place over Zoom. During the informed consent session, trained study staff will first check identification to confirm identity and then go over the informed consent form with prospective participants. During this meeting, interested individuals will be given the opportunity to ask study staff any questions they may have to address concerns and to ensure comprehension among any and all prospective participants. The informed consent form will be programmed into REDCap, and all responses will be stored in a password-protected study folder separate from other study responses (eg, the baseline survey). Only trained study staff will have access to the REDCap responses.

### Intervention

#### Randomization

After participants have provided written informed consent, they will be randomized into an intervention or control group using a randomization plan generator programmed into REDCap [31,32].

#### Optimization Study Design

**Figure 2:**
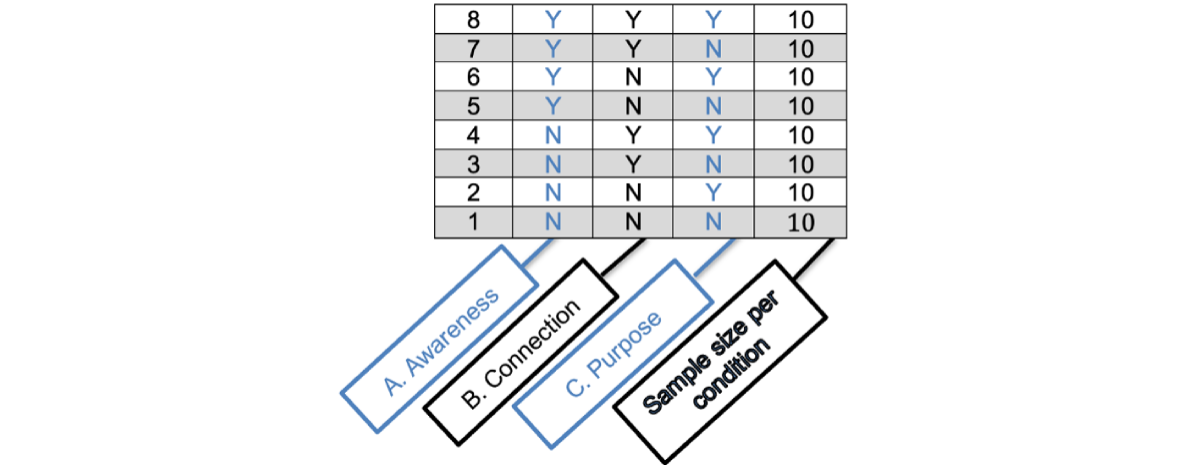
Proposed factorial design for phase 1 intervention optimization.

The factorial design used for this study requires 8 experimental conditions, which were selected and presented in Figure 2. This design should not be considered an RCT because its purpose and logic are different. Although an RCT focuses on the direct comparison between interventions, the present factorial design does not directly compare interventions but rather identifies which components of each intervention are effective at reducing stress and promoting well-being. Following data collection, the main effects and interactions of all 8 conditions will be calculated to estimate efficiency. For instance, the main effect of awareness will be estimated by comparing the mean outcome across conditions 1 to 4 versus the mean outcome across conditions 5 to 8. To calculate the estimate of each main effect accurately, all participants will be included in an intent-to-treat (ITT) analysis. The factorial design does not use a traditional control group as it takes advantage of each factor having 2 levels, one of which serves as a control.

#### Intervention Content

The smartphone app used in this study, HMP, is grounded in neuroscience research [30]. The full HMP app is a self-guided mobile app (available via iOS or Android) that includes the four modules of awareness, connection, insight, and purpose [30,33]. The HMP app was designed by Healthy Minds Innovations, a nonprofit affiliated with the Center for Healthy Minds at the University of Wisconsin in Madison, Wisconsin. Mindfulness meditation is a practice that focuses on an individual’s attention on the present moment, and past research has shown that this can lead to reduced levels of perceived stress [11]. The HMP app consists of prerecorded audio sessions that are designed to be completed over the course of the study (ie, over the 5-day diary period). Each unit is an average of 10 minutes in duration and includes both didactics and guided meditation practices. The participants could access a given unit after they have listened to prior units, and they could go back and replay units if desired. In this study, the app will be activated with a key code so that unit completion and playtime data can be logged for each participant. This study focuses on three aspects of well-being provided through the HMP app: awareness, connection, and purpose. In addition, there is an introductory exercise to mindfulness-based practice that is given to each of the study participants, which serves as the basis of the control group. Each of these components has been shown to reduce stress and promote well-being among populations that experience high rates of discrimination (eg, young SGMs) [34]. The 4 study components are described in detail in subsequent sections.

Introduction to mindfulness-based practice is a 5-minute self-guided introduction to mindfulness practice. This provides information pertaining to breathing and focus; however, information about the three pillars (awareness, connection, and purpose) is not provided during this session. This introduction is given to each intervention condition, and it is the only component given to the control group.

Given the literature demonstrating the benefits of awareness, connection, and purpose, there will be a total of 8 intervention arms for this study representing a combination of these 3 mindfulness-based practices. The eight intervention components are listed in Figure 2: (1) introduction only (ie, the control group); (2) purpose only; (3) connection only; (4) awareness only; (5) purpose and connection; (6) awareness and purpose; (7) awareness and connection; and (8) purpose, awareness, and connection. Daily self-directed mindfulness exercises are expected to last for an average of 10 minutes per day, depending on which intervention the participant is randomized to. However, those who are assigned to the control condition will listen to the introduction to mindfulness practice only and will not engage in self-directed mindfulness exercises each day.

#### Timeline

This study will take place over a 6-day period for each study participant, and the overarching study duration is expected to last 1 year. On the first day, participants will be randomized into 1 of the 8 conditions; complete the baseline survey; and complete the introduction to mindfulness activities or a combination of activities related to awareness, connection, or purpose depending on the randomization. To complete the baseline survey, participants will be sent a hyperlink to complete a baseline survey via REDCap. The baseline survey is expected to last 30-45 minutes. Constructs to be measured during the baseline assessment include, but are not limited to, perceived stress, perceived discrimination, depressive symptoms, and exposure to perceived microaggressions. See Multimedia Appendix 1 Table S1 [35-50] for a full list of the study measures collected during the baseline assessment. In addition, during the informed consent session, participants will be given information pertaining to the HMP app and how to access it. After completing their baseline survey on REDCap, participants will log in to the HMP app and complete their *introduction to mindfulness* exercise, which is expected to last 5 minutes.

On days 2-5, participants will complete their mindfulness activities, which will differ depending on randomization (see the *Intervention* section), which is expected to last between 5 and 15 minutes per day. In addition, participants will be sent a *nightly diary* survey via REDCap that ascertains information pertaining to stressful events and mood states experienced over the course of that day. Stressful events will be measured using the Daily Inventory of Stressful Events (DISE) [51], which is a 7-item questionnaire that ascertains experiences of stress on that day. In addition, if they experienced a stressful event, the DISE asks a follow-up question regarding how stressful the event was for them on a 4-point Likert scale ranging from 1=*not at all* to 4=*very*. To measure mood, participants were given a modified version of the Positive and Negative Affect Schedule (PANAS) in the nightly diary [52]. The PANAS is a 20-item measure that asks respondents to rate how they have felt over the past week (eg, excited or nervous) on a 5-point Likert scale ranging from 1=*very slightly or not at all* to 5=*extremely*. For the purposes of the nightly diary, we modified the PANAS to reflect mood states over the current day. On day 6 of the study, participants will be scheduled for a debriefing session where they will be asked about their experiences in the study and remunerated.

#### Incentives

Participants will have the opportunity to earn up to US $47 for participation in the study, disbursed as a Visa gift card through Giftbit. The financial incentive structure is as follows: (1) participants will receive US $10 to complete their baseline assessment; (2) participants will also be compensated with US $3 for each daily diary completed over the 5-day diary period, up to an additional US $12; (3) participants will receive US $10 for competing their debrief interview. Thus, participants can earn up to US $47 for participation. In addition to the US $47, participants can also earn US $5 for every eligible individual that they recruit into the study who also enrolls.

### Study Outcomes

There are 2 main study outcomes for this study, which are described in detail in subsequent sections.

#### Perceived Stress

Our first study outcome will be the perceived stress of the participants. Participants will be given the Perceived Stress Scale (PSS) [35,53] at both the baseline and follow-up assessments. The PSS is a 10-item measure used to measure the perception of stress over the past month. However, for the purposes of this study, we asked respondents to measure their perceptions of stress over the past day. Responses range on a 5-point Likert scale from 0=*never* to 4=*very often*. An example item is “In the last month, how often have you been upset because of something that happened unexpectedly” [35,53]. Items will be summed to generate a PSS score for both baseline and follow-up, with lower scores denoting less perceived stress over the last month. The Cronbach *α* for the PSS demonstrates high reliability among LGB adults (α=.92) [54].

#### Mental Well-being

Our second study outcome is related to the well-being of the study participants. Participants will be given the Satisfaction with Life Scale (SLS) [36] during both their baseline and follow-up assessments. The SLS is a 5-item measure used to measure an individual’s overall well-being. Responses range on a 7-point Likert scale ranging from 1=*strongly disagree* to 7=*strongly agree*. An example item for the SLS is “The conditions of my life are excellent” [36]. Items will be summed to create a well-being score for both baseline and follow-up, with lower scores denoting lower well-being. The Cronbach α for the SLS has demonstrated acceptable reliability among SGM populations (α=.83) [55].

#### Secondary Study Outcomes

Secondary study outcomes will include mood and stressful events, which will be analyzed via the 5-day nightly diary. Mood will be ascertained via the 20-item PANAS [56]. The PANAS measures positive and negative affect, and 10 items are given for each trait. The PANAS asks respondents to indicate the extent to which they felt a certain way over the past week; however, for the purposes of this study, we asked respondents the extent to which they felt a way over the past day (eg, interested or hostile). Responses lie on a 5-point Likert scale ranging from 1=*very slightly or not at all* to 5=*extremely*. The Cronbach *α* for the PANAS has demonstrated acceptable reliability for both positive and negative affect among LGB populations (*α*=.89 and *α*=.86, respectively) [57]. Exposure to stressful events will be measured via the DISE [51], which asks respondents whether they have experienced 1 of 8 stressors over the past day (eg, argument or disagreement with anyone). If a respondent indicates that they have experienced a given stressor, they are asked follow-up questions pertaining to stressor timing, intensity, perceived stress related to the stressor, resolution (yes or no), and stressor reappraisal. Responses for the follow-up questions lie on a 4-point Likert scale ranging from 0=*none at all* to 3=*very*.

### Data Analysis

#### Univariate Analysis

Frequency tables and summary statistics will be calculated for each variable collected at baseline.

#### Main Study Outcomes Analysis

To assess efficacy, multiple linear regression will be used to estimate the effects of components on the mean change in stress (mean change in the PSS from baseline to follow-up) and well-being (mean change in the SLS). We will use an ITT approach for the analysis. Intervention components will be effect-coded to estimate the main effects and 2-way interactions of all 3 components. To select the optimized intervention, the research team will meet to determine the intervention components that demonstrate empirical evidence of efficacy the strongest or best of which will be considered candidates for the optimized intervention package based on procedures described by Collins et al [58]. Briefly, a component may be unselected if it interacts with another component to the extent that it undermines the effect of the second component.

#### Secondary Study Outcomes Analysis

Multilevel modeling will be used to examine if there are daily-level changes in positive and negative affect, as well as daily stressful events, given the participants’ randomization. We will use an ITT approach for the analysis. We will examine whether there are daily-level changes in positive and negative affect and daily stressors by using a multilevel model that relates the repeated measures of positive and negative affect and daily stressors to the randomization assignment with time entered as a class variable to model potentially nonlinear patterns for 3 models. Furthermore, we will include covariates such as gender identity within these analyses. For normally distributed outcomes, the model will be fit as a linear mixed effects model. For nonnormal outcomes, generalized linear mixed effects modeling will be used.

#### Power Analyses

For the primary outcomes of stress reduction and increased well-being at follow-up, we used the FactorialPowerPlan [59] package in the R software package to estimate the sample size needed for individual main effects of intervention components corresponding to a pretest–posttest correlation of 0.6, a medium effect size as standardized mean difference (Cohen *d*=0.50), given *α*=.05. A sample size of 80 provides 78% power.

## Results

This project was funded in April 2020, and received institutional review board approval on April 27, 2020. After a delay owing to the COVID-19 pandemic, data collection commenced in February 2021. As of December 2021, 60 participants have been enrolled.

## Discussion

This optimization trial is designed to test the efficacy, feasibility, and acceptability of implementing an application-based, mindfulness-based intervention to reduce stress from discrimination and improve well-being among young SGMs of color. We anticipate being able to identify which combination of mindfulness-based components is effective in reducing stress from discrimination. In addition, we will collect several indicators of feasibility and acceptability through retention metrics (eg, completion rates) and a qualitative debrief interview. Overall, this study has the potential to create a sustainable intervention for SGMs of color that uses an innovative mindfulness-based application.

## Appendix

Multimedia Appendix 1 Description of scales used in the baseline assessment.

Multimedia Appendix 2 Peer review report from New York University, Institute of Human Development and Social Change.

## Abbreviations

DISE: Daily Inventory of Stressful Events
EA: emerging adulthood
HMP: Healthy Minds Program
ITT: intent-to-treat
MOST: multiphase optimization strategy
PANAS: Positive and Negative Affect Schedule
PSS: Perceived Stress Scale
RCT: randomized controlled trial
REDUCE: Reduce Stress from Discrimination among Young Sexual and Gender Minorities of Color
SGM: sexual and gender minority
SLS: Satisfaction with Life Scale
SMS: sexual minority stress
