# Supplementary material for: A Mindfulness-Based Intervention to Alleviate Stress From Discrimination Among Young Sexual and Gender Minorities of Color: Protocol for a Pilot Optimization Trial
Source: JMIR Res Protoc. 2022 Jan 14;11(1):e35593. doi: 10.2196/35593 (PMC8800091; doi:10.2196/35593)
Supplement: Multimedia Appendix 1 [file resprot_v11i1e35593_app1.docx]

| **Supplementary Table 1. Baseline assessment measures** | |
| --- | --- |
| **Measure** | **Description** |
| The LGBT People of Color Microaggressions Scale [37] | This scale will be used to assess experiences of, and perceived stress related to, microaggressions experienced from within the LGBT community. This scale has demonstrated excellent internal consistency (α = .92) [37] and is often used as a measure of intersectionality. |
| The Center for Epidemiological Studies Depression Scale (CES-D) [38] | The CES-D is a 20-item measure that ascertains depressive symptoms experienced over the past week [38]. Responses range on a 4-item Likert scale ranging from 1 = *Rarely or none of the time* to 4 = *Most or all of the time*. This scale has demonstrated excellent internal consistency among LGB samples (e.g., α = .93) [39]. |
| Perceived Stress Scale (PSS) [35] | The PSS is a 14-item measure that examines feelings of perceived stress over the past month. Responses range on a 5-point Likert scale ranging from 0 = *Never* to 4 = *Very often*. This scale has demonstrated acceptable internal consistency among LGB samples (e.g., α = .87) [40]. |
| Satisfaction with Life Scale (SWLS) [36] | The SWLS is a 5-item measure that measures global life satisfaction [36]. Responses range on a 7-point Likert scale ranging from 1 = *Strongly disagree* to 7 = *Strongly agree*, with higher scores denoting greater satisfaction with life. The SWLS has demonstrated good internal consistency [36]. |
| Warwick-Edinburgh Mental Well-being Scale (WEMWBS) [41] | The WEMWBS is a 14-item measure that measures mental well-being over the past two weeks [41]. Responses are on a 5-point Likert scale ranging from 1 = *None of the time* to 5 = *All of the time*, with higher scores denoting better mental well-being. This scale has demonstrated excellent internal consistency among SGM (α = .92) [42]. |
| Subjective Happiness Scale (SHS) [43] | The SHS is a 4-item scale that measures overall feelings of happiness. Responses lie on a 7-point Likert scale, with higher responses denoting greater feelings of happiness. This scale has demonstrated good internal consistency ranging from .79 to .94 [43]. |
| Distress Tolerance Scale (DTS) [44] | The DTS is a 15-item measure that measures one’s ability to tolerate stress [44]. Responses lie on a 5-point Likert scale ranging from 1 = *Strongly agree* to 5 = *Strongly disagree*, with higher scores denoting greater tolerance to stress. This scale has demonstrated good internal consistency among LGB adults (α = .92) [45]. |
| Barratt Impulsiveness Scale (BIS-11) [46] | The BIS-11 is a 30-item scale that measures one’s tendency towards impulsiveness [46]. Responses are on a 4-point Likert scale ranging from 1 = *Rarely/never* to 4 = *Almost always/always*, with higher scores denoting greater impulsiveness. This scale has demonstrated acceptable internal consistency among LGBT youth (α = .76) [47]. |
| The Penn State Worry Questionnaire (PSWQ) [48] | The PSWQ is a 16-item measure that ascertains one’s tendency to worry [48]. Responses range on a 5-point Likert scale ranging from 1 = *Not at all typical of me* to 5 = *Very typical of me*, with higher scores denoting a greater tendency to worry. This scale has shown excellent internal consistency (α = .95) [48]. |
| Coping with Discrimination Scale (CDS) [49] | The CDS is a 25-item measure that examines one’s ability to cope with experiences of discrimination [49]. Responses range on a 5-point Likert scale, with higher scores suggesting a better ability to cope with experiences of discrimination. The CDS has shown adequate internal consistency among sexual minority adults [50]. |

**References**

35. Cohen S, Kamarck T, Mermelstein R. A Global Measure of Perceived Stress. Journal of Health and Social Behavior. 1983;24(4):385-96.

36. Diener E, Emmons RA, Larsen RJ, Griffin S. The Satisfaction With Life Scale. Journal of Personality Assessment. 1985;49(1):71-5.

37. Balsam KF, Molina Y, Beadnell B, Simoni J, Walters K. Measuring multiple minority stress: The LGBT People of Color Microaggressions Scale. Cultural Diversity and Ethnic Minority Psychology. 2011;17(2):163-74.

38. Radloff LS. The CES-D Scale: A Self-Report Depression Scale for Research in the General Population. Applied Psychological Measurement. 1977;1(3):385-401.

39. McCarthy MA, Fisher CM, Irwin JA, Coleman JD, Pelster ADK. Using the Minority Stress Model to Understand Depression in Lesbian, Gay, Bisexual, and Transgender Individuals in Nebraska. Journal of Gay & Lesbian Mental Health. 2014;18(4):346-60.

40. Tatum AK, Ross MW. A longitudinal analysis of sexual minorities’ acceptance concerns and internalised homonegativity on perceived psychological stress. Psychology & Sexuality. 2020:1-13.

41. Tennant R, Hiller L, Fishwick R, Platt S, Joseph S, Weich S, et al. The Warwick-Edinburgh Mental Well-being Scale (WEMWBS): development and UK validation. Health Qual Life Outcomes. 2007;5:63.

42. Hunter J, Butler C, Cooper K. Gender minority stress in trans and gender diverse adolescents and young people. Clinical Child Psychology and Psychiatry. 2021:13591045211033187.

43. Lyubomirsky S, Lepper HS. A Measure of Subjective Happiness: Preliminary Reliability and Construct Validation. Social Indicators Research. 1999;46(2):137-55.

44. Simons JS, Gaher RM. The Distress Tolerance Scale: Development and Validation of a Self-Report Measure. Motivation and Emotion. 2005;29(2):83-102.

45. Reitzel LR, Smith NG, Obasi EM, Forney M, Leventhal AM. Perceived distress tolerance accounts for the covariance between discrimination experiences and anxiety symptoms among sexual minority adults. Journal of Anxiety Disorders. 2017;48:22-7.

46. Patton JH, Stanford MS, Barratt ES. Factor structure of the barratt impulsiveness scale. Journal of Clinical Psychology. 1995;51(6):768-74.

47. Liu RT, Mustanski B. Suicidal Ideation and Self-Harm in Lesbian, Gay, Bisexual, and Transgender Youth. American Journal of Preventive Medicine. 2012;42(3):221-8.

48. Meyer TJ, Miller ML, Metzger RL, Borkovec TD. Development and validation of the penn state worry questionnaire. Behaviour Research and Therapy. 1990;28(6):487-95.

49. Wei M, Alvarez AN, Ku T-Y, Russell DW, Bonett DG. Development and validation of a Coping with Discrimination Scale: Factor structure, reliability, and validity. Journal of Counseling Psychology. 2010;57(3):328-44.

50. Ngamake ST, Walch SE, Raveepatarakul J. Validation of the Coping With Discrimination Scale in Sexual Minorities. Journal of Homosexuality. 2014;61(7):1003-24.
