# Supplementary material for: A Mindfulness-Based Intervention to Alleviate Stress From Discrimination Among Young Sexual and Gender Minorities of Color: Protocol for a Pilot Optimization Trial
Source: JMIR Res Protoc. 2022 Jan 14;11(1):e35593. doi: 10.2196/35593 (PMC8800091; doi:10.2196/35593)
Supplement: Multimedia Appendix 2 [file resprot_v11i1e35593_app2.pdf]

Institute of **Human Development** and **Social Change**

New York University
Steinhardt School of Culture, Education, and Human Development
Robert F. Wagner Graduate School of Public Service
Graduate School of Arts and Science

Director: Elise Cappella

February 13th, 2020

Dear Stephanie, Shabnam, and Erin,

Thank you for participating in the IHDSC 2019-2020 Seed Award competition. Your application entitled "Optimizaing a Daily Mindfulness Intervention to Reduce Stress from Discrimination among Sexual and Gender Minorities of Color" has gone through initial review, and we would like to request that you respond to reviewer comments before final decisions are made.

Your proposal was reviewed by an interdisciplinary committee of NYU faculty, and reviewers overwhelmingly felt that it was well aligned with the Institute's mission and funding priorities. Committee members also commented on the strength of the interdisciplinary team, the interesting and novel methods proposed, the potential for findings to yield data with significant implications for public health intervention, and the likelihood that study results would be of interest to both academic and practitioner audiences.

Before a final decision can be made, we request that you respond to the following questions and concerns raised by reviewers:

- Reviewers expressed some concern about plans to recruit and retain 160 difficult-to-engage participants. While the proposal notes that the PI has successfully recruited this type of sample in previous work, it would be helpful to know if they have done so with this population in such a narrow window of time. Please also elaborate on how the project would deal with attrition.
- Please clarify whether there will be any consideration of cultural appropriateness of the mindfulness intervention for this population.
- Reviewers expressed concern about the work given the state of the literature on mindfulness-based approaches for the population. The proposal focuses on optimizing treatment, however the evidence that mindfulness-based intervention is, as a package, effective for similar populations, is unclear. The proposal cites several studies, but these are non-experimental studies. Please provide additional detail regarding the state of the treatment literature for mindfulness based interventions for this population (i.e., details of the preparation phase and its results would be helpful).
- Please provide additional information about dosage. It appears that the intervention lasts five days, which may not be appropriate dosage for the effects of mindfulness to be clearly observed or differences between components to be detected.
- Due to availability of funding, we will only be able to offer awards totaling $15,000. Please provide a revised budget that reflects a total of $15,000. Please also address the following issue regarding the budget:

NEW YORK UNIVERSITY

246 Greene St 6E | New York, New York 10003-9502
212 992 7695 | 212 998 4718 fax | ihdsc@nyu.edu | www.steinhardt.nyu.edu/ihdsc

- The Participant Incentives section includes a cost of $5,800, but the section total indicates a total of $9,280. According to the budget justification, it appears that $9,280 is the correct amount.

We ask that you provide responses by March 2nd, 2020. Narrative responses can be provided in a brief 1-2 page memo, while revisions to the budget can be submitted as a separate file. Please also feel free to include a separate list of additional references. A final award determination will be made following the receipt of these materials.

Thank you again for submitting a proposal. We look forward to receiving your responses.

Sincerely,

Chris Barker
Assistant Director, Program Development

Addressing Review Comments

We would like to thank the reviewers for their support and insightful comments of our IHDSC Seed application. Below we have responded to all the issues raised by the reviewers.

1. **Reviewers expressed some concern about plans to recruit and retain 160 difficult-to-engage participants. While the proposal notes that the PI has successfully recruited this type of sample in previous work, it would be helpful to know if they have done so with this population in such a narrow window of time. Please also elaborate on how the project would deal with attrition.**

To meet budget and feasibility concerns, we have changed our proposed sample size to be 80 instead of 160 (see response to Comment 2). Given the previous work of the study team in recruiting and maintaining SGM of color in research studies, we believe that the recruitment of n=80 SGM of color is entirely possible within the grant timeline. In addition, with the reduction in conditions, we still have significant power to detect a significant finding.

2. **Due to availability of funding, we will only be able to offer awards totaling $15,000. Please provide a revised budget that reflects a total of $15,000. Please also address the following issue regarding the budget:**

   The Participant Incentives section includes a cost of $5,800, but the section total indicates a total of $9,280. According to the budget justification, it appears that $9,280 is the correct amount.

To bring the budget under 15K and to further support the feasibility of the project in terms of recruiting and retaining SGM of color within the time allotted, we are slightly modifying our design to be a 2^3 (instead of a 2^4). The proposed sample size is now 80 instead of 160. Each condition will have 10 participants. This design supports attrition (we can have about 3 non-completers per group and still have valid pilot results). We will remove the "Purpose" component because out of all of the components it has the least amount of supportive evidence. However, we will re-include this in the larger grant proposal. The goal of this pilot is to examine feasibility and acceptability, so even with the proposed modifications, we strongly believe that the pilot is scientifically rigorous and will lead to a successful R01 grant application. Please see the updated budget and justification.

3. **Please clarify whether there will be any consideration of cultural appropriateness of the mindfulness intervention for this population.**

This is a very important question for which we continue to grapple. We have decided not to alter the content of the application at this stage. Indeed, there is much heterogeneity within SGM of color. However, we have pilot tested the application with 10 different SGM of color and received positive feedback, including that the application and the included modules are relevant and acceptable. Small comments concerning the font and background color arose during the preparation phase; however, these are easily addressable. It is vitally important that we optimize an intervention that is sustainable and can be rolled out immediately. Inherent in MOST is the idea that we are not looking for the “best” intervention package. This would, financially speaking, not be obtainable. Instead, we want to create an intervention package that is effective, cost-effective, can reach the most SGM of color, and can be immediately distributed to an in-need community. To this end, we continue to be mindful of ways that we may need to tailor the application in the future, and indeed, we ask debrief questions concerning this matter. However, based on our initial piloting of the application and the goal of this pilot study we do not believe

there are any other needed modifications to the application with respect to cultural appropriateness. Based on the findings of this pilot study, we will bring together the research team and our community partners to discuss how to modify the application for different cultural realities of SGM of color, if need be.

4. **Reviewers expressed concern about the work given the state of the literature on mindfulness-based approaches for the population. The proposal focuses on optimizing treatment, however the evidence that mindfulness-based intervention is, as a package, effective for similar populations, is unclear. The proposal cites several studies, but these are non-experimental studies. Please provide additional detail regarding the state of the treatment literature for mindfulness-based interventions for this population (i.e., details of the preparation phase and its results would be helpful).**

There is an overwhelming amount of research literature showing the effectiveness of mindfulness-based interventions for vulnerable populations.[1-10] This research includes randomized clinical trials, observational studies, etc. This underlying body of research suggests that that mindfulness-based interventions should also be effective among SGM of color. However, there is also substantive work showing that mindfulness-based interventions are effective among SGM.[11] For instance, Lyons[12] found in a study of older gay men who experienced psychological distress and low self-esteem from experiences of discrimination that dispositional mindfulness attenuated the mental health impact of sexuality- and age-related discrimination on psychological distress. Further, researchers found in an RCT of gay HIV positive men, that in comparison to the control group, the group that received the mindfulness-based stress reduction (MBSR) had greater positive affect at the 8-week and 6-month follow up visits.[13] Further, in a pilot RCT of rural LGB individuals, researchers found that perceived stress decreased by 23% in women and by 40% in men between baseline and follow up. Women demonstrated a 12% reduction in overall minority from baseline to 12-week follow-up.[14] Taken together, observational studies as well as RCTs suggest that mindfulness-based interventions among LGB work and may be vitally important to reducing the negative effects of stress specific to these populations. In addition to the work described, there has been an overall paradigm shift within the community to think more critically about the role of mindfulness as a meaningful and valuable intervention tool. This is evidenced by the usage of mindfulness in the ESTEEM RCT[7] currently underway and a recent review article outlining the importance of mindfulness for LGB.[11] In this specific proposal, we are building upon this work by attempting to build a feasible and acceptable application-based mindfulness intervention that may can be immediately delivered to SGM communities through our partners Healthy Minds Inc.

5. **Please provide additional information about dosage. It appears that the intervention lasts five days, which may not be appropriate dosage for the effects of mindfulness to be clearly observed or differences between components to be detected.**

As part of our pilot we will be assessing feasibility in terms of the timing/dosage of the intervention. However, we are confident that we are starting at the correct dosage. Pogrebtsova et al.[15] found in a similar daily diary RCT that negative affect declined over 5-days compared to the control groups. Thus, in our 5-day daily diary optimization trial we expect to see statistically significant results or results trending towards statistical significance. In addition, in our debrief interview we also assess participant burden by asking questions related to the timing of the intervention (e.g. "was 5-days enough time to fully connect with the intervention?"). If we find that the 5-day daily diary is not enough time, we will utilize this as evidence to extend timing in our R01 submission.

References

1. Chiesa A, Serretti A. Mindfulness-based interventions for chronic pain: A systematic review of the evidence. *The Journal of Alternative and Complementary Medicine.* 2011;17(1):83-93.
2. Chiesa A, Serretti A. Are mindfulness-based interventions effective for substance use disorders? A systematic review of the evidence. *Substance Use & Misuse.* 2014;49(5):492-512.
3. Felver JC, Celis-de Hoyos CE, Tezanos K, Singh NN. A systematic review of mindfulness-based interventions for youth in school settings. *Mindfulness.* 2016;7(1):34-45.
4. Goldberg SB, Tucker RP, Greene PA, et al. Mindfulness-based interventions for psychiatric disorders: A systematic review and meta-analysis. *Clinical Psychology Review.* 2018;59:52-60.
5. Gotink RA, Chu P, Busschbach JJ, Benson H, Fricchione GL, Hunink MM. Standardised mindfulness-based interventions in healthcare: an overview of systematic reviews and meta-analyses of RCTs. *PloS One.* 2015;10(4):e0124344.
6. Kabat-Zinn J. Mindfulness-based interventions in context: past, present, and future. *Clinical Psychology: Science and Practice.* 2003;10(2):144-156.
7. O'Reilly GA, Cook L, Spruijt-Metz D, Black DS. Mindfulness-based interventions for obesity-related eating behaviours: a literature review. *Obesity Reviews.* 2014;15(6):453-461.
8. Spijkerman M, Pots WTM, Bohlmeijer ET. Effectiveness of online mindfulness-based interventions in improving mental health: A review and meta-analysis of randomised controlled trials. *Clinical Psychology Review.* 2016;45:102-114.
9. Veehof M, Trompetter H, Bohlmeijer ET, Schreurs KMG. Acceptance-and mindfulness-based interventions for the treatment of chronic pain: a meta-analytic review. *Cognitive Behaviour Therapy.* 2016;45(1):5-31.
10. Zenner C, Herrnleben-Kurz S, Walach H. Mindfulness-based interventions in schools—a systematic review and meta-analysis. *Frontiers in Psychology.* 2014;5:603.
11. Iacono G. An Affirmative Mindfulness Approach for Lesbian, Gay, Bisexual, Transgender, and Queer Youth Mental Health. *Clinical Social Work Journal.* 2019;47(2):156-166.
12. Lyons A. Mindfulness attenuates the impact of discrimination on the mental health of middle-aged and older gay men. *Psychology of Sexual Orientation and Gender Diversity.* 2016;3(2):227-235.
13. Gayner B, Esplen MJ, DeRoche P, et al. A randomized controlled trial of mindfulness-based stress reduction to manage affective symptoms and improve quality of life in gay men living with HIV. *Journal of Behavioral Medicine.* 2012;35(3):272-285.
14. Tree JMJ, Patterson JG. A Test of Feasibility and Acceptability of Online Mindfulness-Based Stress Reduction for Lesbian, Gay, and Bisexual Women and Men at Risk for High Stress: Pilot Study. *JMIR Mental Health.* 2019;6(8):e15048.
15. Pogrebtsova E, Craig J, Chris A, O'shea D, González-Morales MG. Exploring daily affective changes in university students with a mindful positive reappraisal intervention: A daily diary randomized controlled trial. *Stress and Health.* 2018;34(1):46-58.
